# Nerve injury inhibits *Oprd1* and *Cnr1* transcription through REST in primary sensory neurons

**DOI:** 10.1038/s41598-024-74487-1

**Published:** 2024-11-04

**Authors:** Ashok Subedi, Asieh Etemad, Aadhya Tiwari, Yuying Huang, Biji Chatterjee, Samantha M. McLeod, Yungang Lu, DiAngelo Gonzalez, Krishna Ghosh, Mario Sirito, Sanjay K. Singh, Elisa Ruiz, Sandra L. Grimm, Cristian Coarfa, Hui-Lin Pan, Sadhan Majumder

**Affiliations:** 1 Department of Genetics, The University of Texas MD Anderson Cancer Center, 77030 Houston, TX USA; 2 Department of Anesthesiology & Perioperative Medicine, The University of Texas MD Anderson Cancer Center, 77030 Houston, TX USA; 3 Department of Neurosurgery, The University of Texas MD Anderson Cancer Center, 77030 Houston, TX USA; 4 Department of Molecular and Cell Biology, Baylor College of Medicine, 77030 Houston, TX USA; 5 Present Address: Baylor College of Medicine, 77030 Houston, TX USA

**Keywords:** REST, Dorsal root ganglion, Chronic pain, Opioid receptors, Cannabinoid CB1 receptor, Therapeutic target, Neuroscience, Chronic pain

## Abstract

The transcription repressor REST in the dorsal root ganglion (DRG) is upregulated by peripheral nerve injury and promotes the development of chronic pain. However, the genes targeted by REST in neuropathic pain development remain unclear. The expression levels of four opioid receptor genes (*Oprm1*, *Oprd1*, *Oprl1* and *Oprk1*) and the cannabinoid CB1 receptor (*Cnr1*) gene in the DRG regulate nociception. In this study, we determined the role of REST in controlling their expression in the DRG induced by spared nerve injury (SNI). SNI induced chronic pain hypersensitivity in wild-type mice and was accompanied by increased levels of *Rest* transcript and protein. Transcriptomic analyses of wild-type mouse DRGs suggested that SNI upregulates the expression of *Rest* transcripts and downregulates the transcripts of all four opioid receptor genes and the *Cnr1* gene. Quantitative reverse transcription polymerase chain reaction analyses of these tissues validated these results. Analysis of publicly available bioinformatic data suggested that REST binds to the promoter regions of *Oprm1* and *Cnr1*. Chromatin immunoprecipitation analyses indicated the presence of REST at these promoters. Full-length *Rest* conditional knockout in primary sensory neurons reduced SNI-induced pain hypersensitivity and rescued the SNI-induced reduction in the expression of *Oprd1* and *Cnr1* in mouse DRG. Our results suggest that nerve injury represses the transcription of at least the *Oprd1* and *Cnr1* genes via REST in primary sensory neurons and that REST is a potential therapeutic target for neuropathic pain. Thus, inhibiting REST activity could potentially reduce chronic neuropathic pain and augment opioid/cannabinoid analgesic actions by increasing the transcription of *Oprd1* and *Cnr1* genes in DRG neurons.

## Introduction

Chronic neuropathic pain is difficult to treat and remains a major clinical problem, adversely affecting the quality of life of millions of Americans^1–4^. The mechanisms underlying the transition from acute to chronic pain after nerve injury remain unclear, but mounting evidence suggests that epigenomic mechanisms (an array of modifications, including DNA methylation and histone marks) play a major role in this transition^5,3^. Persistent changes in the upregulation of pro-nociceptive genes and downregulation of anti-nociceptive genes account for the long-lasting aberrant excitability of dorsal root ganglion (DRG) neurons and excitatory nociceptive input to the spinal cord^6–8^. For example, nerve injury causes upregulation of α2δ-1 in the DRG, which leads to increased synaptic NMDA receptor activity and glutamatergic input to spinal dorsal horn neurons^9,10^ Furthermore, nerve injury reduces the expression of almost all voltage-gated K^+^ channels^11,7,12,13^ contributing to the increased excitability of DRG neurons. Histone methyltransferase G9a-mediated epigenetic regulation in DRG neurons is also essential for the acute-to-chronic pain transition after nerve injury^7,14^.

Opioids, the most powerful analgesics, act through opioid receptors, which belong to the G-protein coupled receptor family (MOR for µ, KOR for κ, DOR for δ, NOP for nociceptin/orphan FQ or N/OFQ)^15^. However, chronic opioid treatment causes analgesic tolerance/hyperalgesia, which has resulted in an opioid addiction epidemic in the U.S. The various opioid receptors have been shown to have diverse roles in opioid sensitivity and the development of opioid tolerance. In this regard, opioid-induced analgesia and hyperalgesia/analgesic tolerance depend critically on the expression level of O*prm1*, which encodes the µ-opioid receptor, in DRG neurons^16^. Also, the δ-opioid receptor (DOR) in primary sensory neurons regulates the analgesic effect of DOR agonists but not morphine-induced analgesic tolerance^17^. Nerve injury in mice reduces opioid analgesia via G9a-mediated repression of *Oprm1*^18^. G9a also suppresses cannabinoid CB1 receptor (encoded by *Cnr1*) expression in primary sensory neurons^19^. Indeed, studies have suggested that the four opioid receptor genes and the cannabinoid CB1 receptor (*Cnr1*) gene in the DRG play a role in regulating nociception^3^. In addition, peripheral nerve injury causes overexpression of the transcriptional repressor RE1-silencing transcription factor (REST, also known as Neuron *Rest*rictive Silencer Factor)^20–22^ in the DRG of male mice, which induces the repression of REST-targeted genes such as such as *Kcnd3*,* Kcnq2*,* Scn10a*,* Chrm2*, and the *Oprm1*^23,24^. However, it remains unclear whether REST controls the expression of the 4 opioid receptor and CB1 receptor genes in the DRG.

All previous studies of REST-mediated chronic pain^25,24^ were performed using a *Rest* conditional knockout (cKO) mouse model that has now been discovered to harbor only partial *Rest* cKO^22^ with only ~ 40% loss of REST activity, generating confusion in some areas of research on primary sensory neurons. The Mandel lab then created a highly robust full-length *Rest* cKO mouse line^22^, which was used in the present study. To our knowledge, this would be the first use of the full-length *Rest* cKO mouse line in a chronic pain study. Here we studied the role of REST in nerve injury-induced chronic pain in controlling the expression of opioid receptor and cannabinoid CB1 receptor genes in DRG neurons in mice.

## Results

### Peripheral nerve injury induces chronic pain hypersensitivity in wild-type mice

**Fig. 1:**
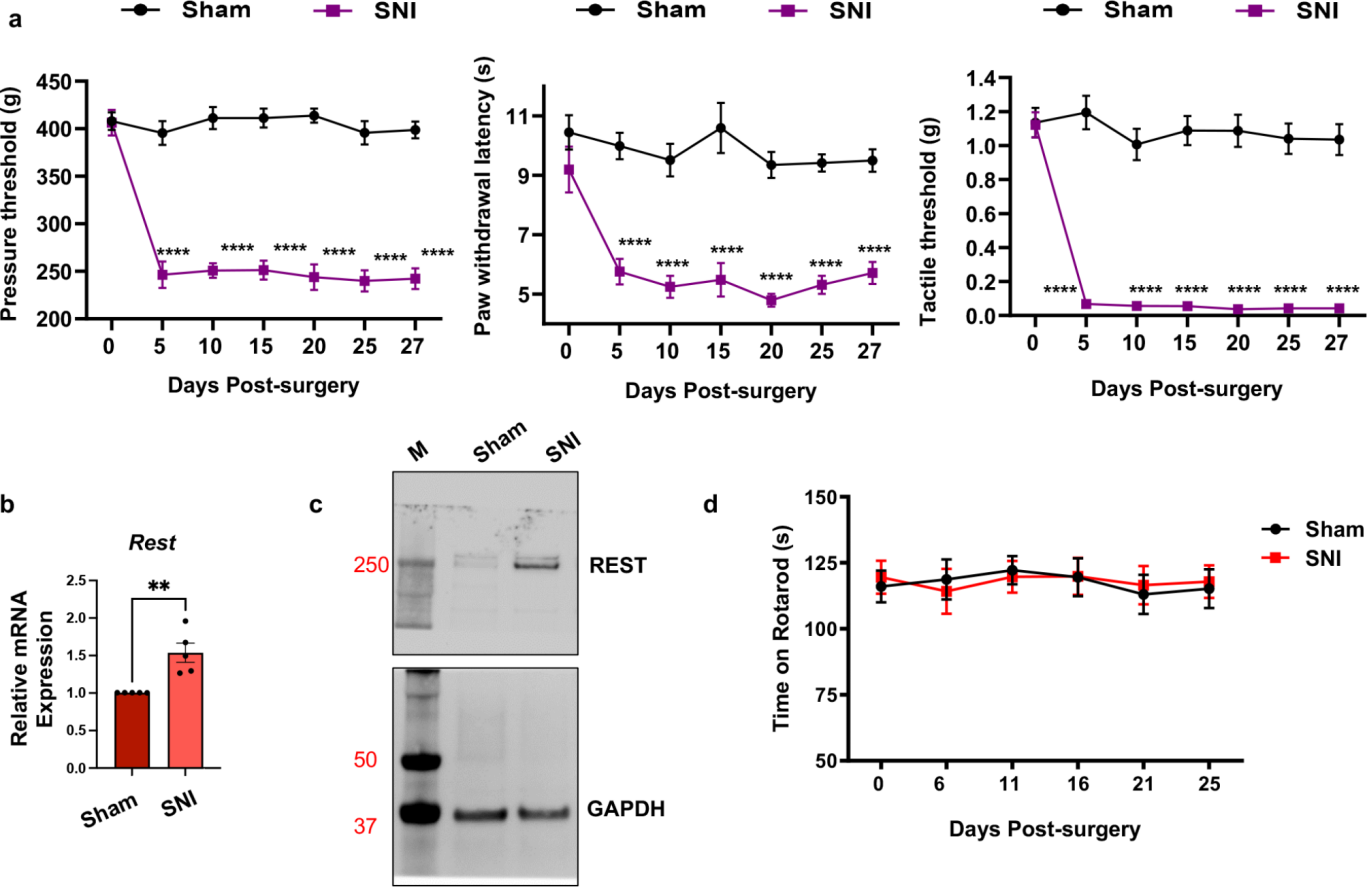
(**a**) Peripheral nerve injury induces chronic neuropathic pain hypersensitivities in wild-type mice. Either spared nerve injury (SNI) or sham surgery (Sham) was performed on the left hind limbs of male wild-type mice. Pain symptoms, in terms of ipsilateral hindpaw withdrawal thresholds (withdrawal latency), were determined in response to a noxious pressure stimulus (mechanical hyperalgesia), noxious radiant heat (thermal hyperalgesia), and von Frey filaments (tactile allodynia) over 27 days (Days post-surgery). *N* = 6 per group. A Student’s t-test was used. P-values are shown (* <0.05; **<0.01; ***<0.001; ****<0.0001) compared with sham group. Data are presented as mean ± SEM. (**b**–**d**) SNI results in increased mRNA and protein levels of the REST gene in the injured DRG of wild-type mice, but it does not cause significant changes in the rotarod assays. (**b**) mRNA samples from the L3/L4 DRG tissues of mice, characterized in Fig. 1a, were used for RT-qPCR analysis of *Rest* transcripts. *Gapdh* was used as an internal control. A Student’s t-test was used to measure the p-value. *N* = 5 mice per group. Data are presented as mean ± SEM. (**c**) Western blotting assays from the L3/L4 DRG tissues indicated a corresponding increase in the REST protein. M = Molecular weight ladder. Approximate molecular weights in kilodalton are also shown. (**d**) Rotarod assays of wild-type mice indicate no significant difference between sham and SNI treatment.

To establish a mouse model of neuropathic pain development, we performed either spared nerve injury (SNI) or sham surgery in male wild-type C57BL/6 mice (Fig. 1a). We then measured ipsilateral hindpaw withdrawal thresholds in response to a noxious pressure stimulus (mechanical hyperalgesia), noxious radiant heat (thermal hyperalgesia), and von Frey filaments (tactile allodynia) for four weeks. SNI induced reductions in the withdrawal thresholds in the injured hindpaws of these mice. Mechanical, thermal, and tactile hypersensitivity developed within five days and persisted for at least four weeks.

As described above, peripheral nerve injury causes overexpression of REST^23,25,24^. To determine whether our experimental SNI method recapitulated this observation, we collected ipsilateral DRG tissues at the L3/L4 level 28 days after SNI or sham surgery from individual mice, as shown in Fig. 1a. We then extracted mRNA from the tissues and used RT-qPCR to analyze the mRNA levels of *Rest*. We found that *Rest* transcript levels were increased in these mice (Fig. 1b). In addition, Western blotting assays from the DRG tissues also indicated a corresponding increase in the REST protein (Fig. 1c; raw data of the Western blot is shown in Supplementary Fig. 1).

Because we measured evoked pain hypersensitivity using a reflex response, we performed rotarod tests to determine whether SNI induces impairment of motor functions. As shown in Fig. 1d, there were no significant differences in the rotarod performance tests between mice treated with SNI and those that underwent sham surgery, suggesting that SNI did not diminish motor functions. Thus, our system reproduced the chronic pain behavior anticipated based on the work of previous researchers in the field.

### Nerve injury leads to the transcriptomic activation of Rest and the repression of opioid receptor genes and the cannabinoid receptor gene in the injured DRG of wild-type mice

Previous studies have shown that nerve injury impacts the expression of *Oprm1*, *Oprd1*, and *Cnr1* in the DRG^19,26,16^. To determine whether other opioid receptors are also impacted by nerve injury, we collected ipsilateral DRG tissues at the L3/L4 level 28 days after SNI or sham surgery from three individual male mouse, as shown in Fig. 1, and performed RNA sequencing. We analyzed the data as described previously^27–29^.

**Fig. 2:**
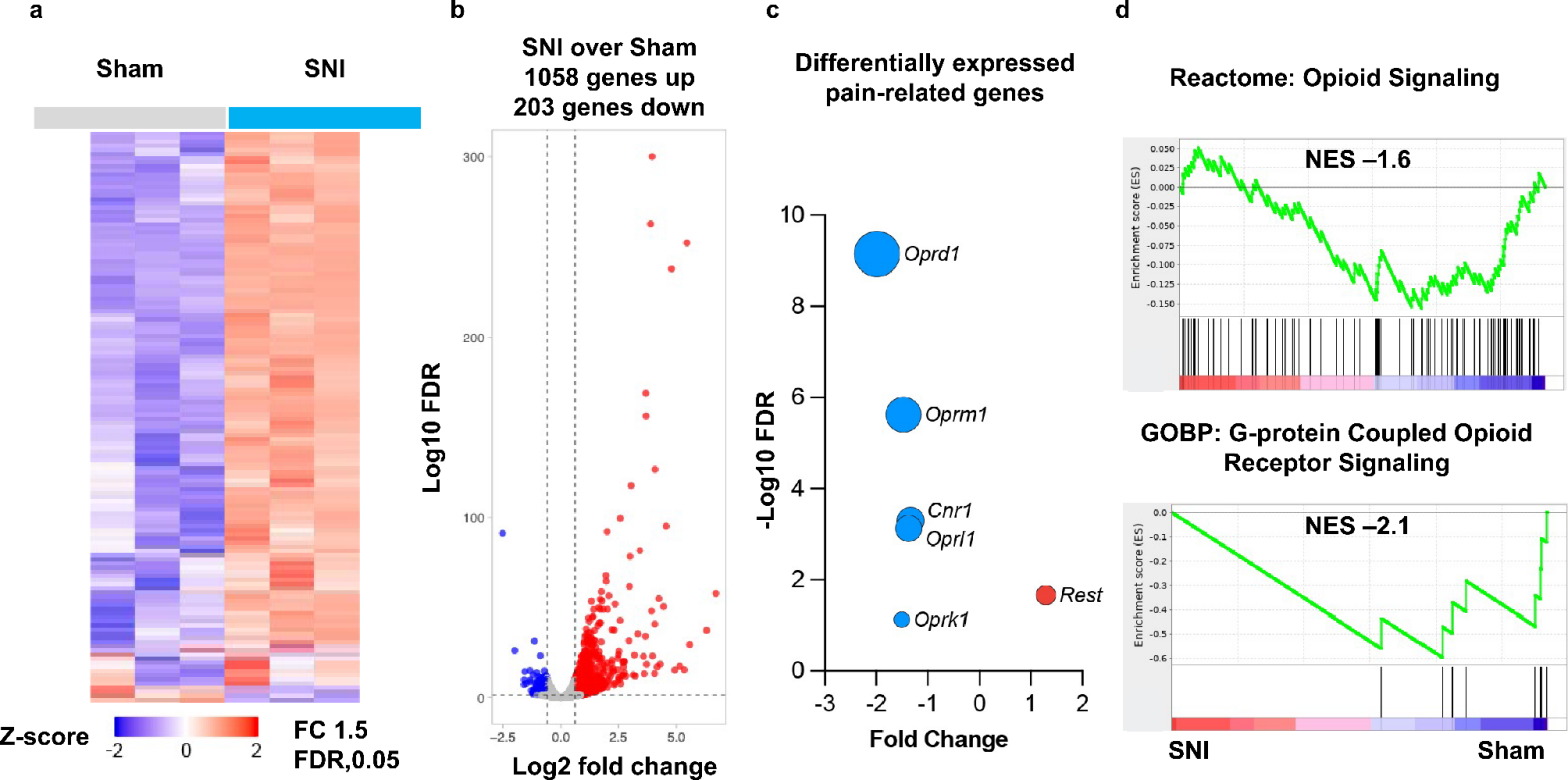
SNI leads to transcriptomic activation of *Rest* and repression of opioid receptor and cannabinoid CB1 receptor genes in the injured DRG of wild-type mice. mRNA samples from the L3/L4 DRG tissues of 3 individual male mice were examined by RNA-Seq analyses. (**a**) Hierarchical clustering of differentially expressed genes between SNI and Sham surgery groups. (**b**) Volcano plot showing differentially expressed genes. (**c**) Differential expression analysis of *Rest*, *Oprd1*, *Oprk1*, *Oprl1*, *Oprm1*, and *Cnr1* between SNI and Sham surgery groups. The size of the circle is -log10(FDR). (**d**) Gene Set Enrichment Analysis shows downregulation of the opioid signaling pathway. Experiments were performed in triplicate. NES represents Normalized Enrichment Score.

Hierarchical clustering of differentially expressed genes between SNI and Sham surgery groups suggested differential expression of genes between these two groups (Fig. 2a; See also Supplementary Table 1). Volcano plots showed significant upregulation of 1058 genes and downregulation of 203 genes in the injured DRG (Fig. 2b). We focused on the expression levels of four opioid receptor genes (*Oprm1*, *Oprd1*, *Oprl1*, *Oprk1*) and the cannabinoid CB1 receptor (*Cnr1*) gene. Differential expression analysis of these genes suggested that while the *Rest* gene transcript was induced by SNI, as expected from our results shown in Fig. 1b, the opioid receptor genes *Oprd1*, *Oprm1*, *Oprl1*, and *Oprk1*, as well as the cannabinoid CB1 receptor gene *Cnr1*, were suppressed (Fig. 2c). Interestingly, only *Oprd1* was significant at FDR < 0.05 and had a fold change exceeding 1.5x, hence only that gene was presented in Supplementary Table 1. *Oprm1*, *Cnr1*, *Oprl1*, and *Rest* were also significant with an FDR < 0.05, but their fold changes were below 1.5x. In contrast, the gene *Oprk1* had an FDR of 0.0765 and a fold change greater than 1.5x. Supplementary Table 2 displays the Log2 fold change, linear fold change, p-value, and FDR for the genes *Rest*, *Oprd1*, *Oprk1*, *Oprl1*, *Oprm1*, and *Cnr1*. Gene Set Enrichment Analysis indicated a trend suggesting the suppression of opioid signaling and G-protein coupled opioid receptor signaling pathways (Fig. 2d), which corroborates this observation. These results suggest that nerve injury represses the expression of all four opioid receptor genes and the *Cnr1* gene in the DRG of mice.

### SNI decreases mRNA levels of opioid receptor genes and the cannabinoid CB1 receptor gene in the DRG of wild-type mice

**Fig. 3:**
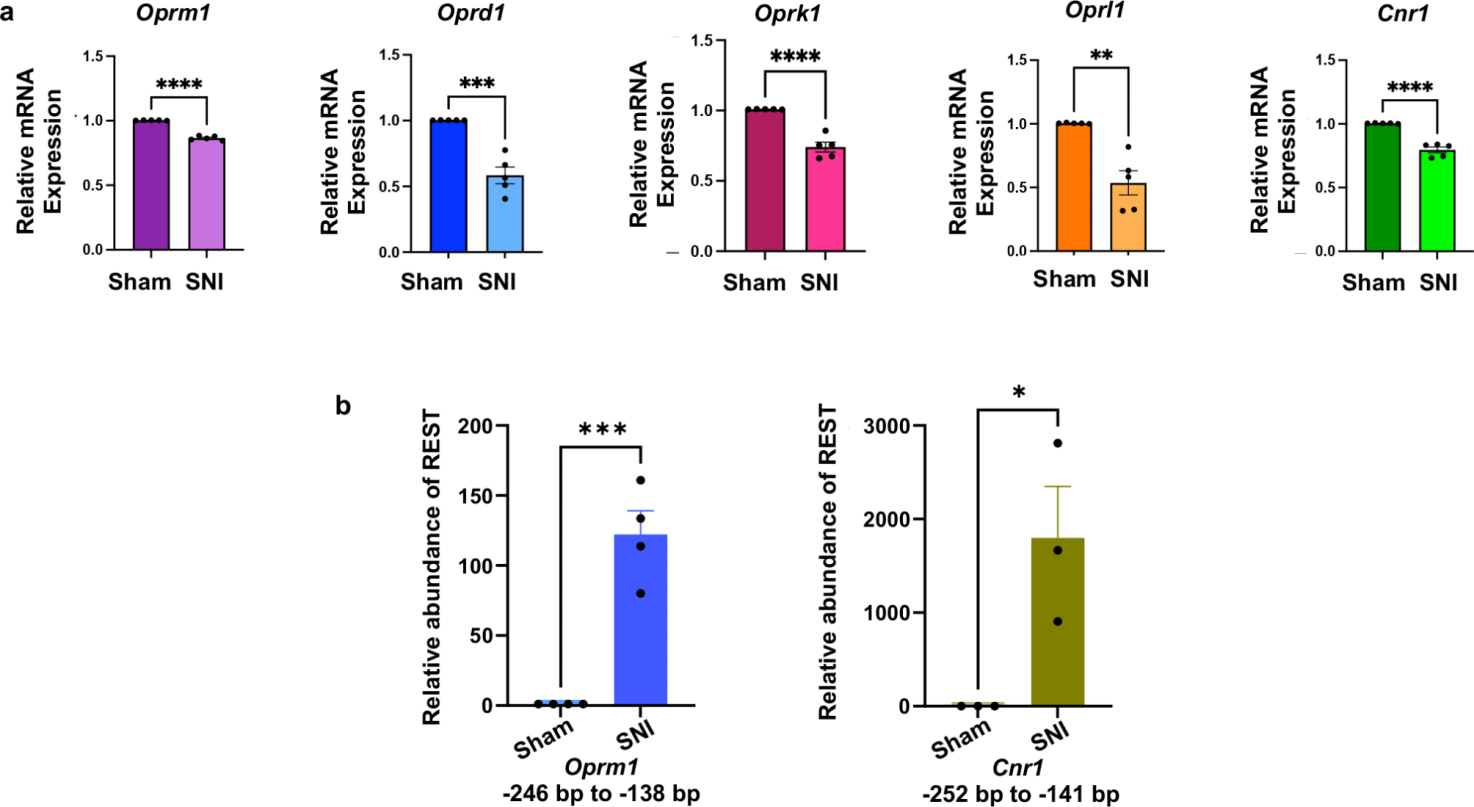
(**a**) SNI results in decreased mRNA levels of opioid receptor and cannabinoid CB1 receptor genes in the injured DRG of wild-type mice. mRNA samples from the L3/L4 DRG tissues of mice, characterized in Fig. 1, were used for RT-qPCR analysis of *Oprm1*, *Oprd1*, *Oprk1*, *Oprl1*, and *Cnr1* transcripts. *Gapdh* was used as an internal control. A Student’s t-test was used to measure the p-value. *N* = 5 mice per group. Data are presented as mean ± SEM. (**b**) REST binds to the *Oprm1* and *Cnr1* promoters in the injured DRG of wild-type mice. REST binding to *Oprm1* and *Cnr1* promoters was measured by qChIP assay in sham and SNI in mice. *N* = 3 mice per group. A Student’s t-test was used to measure the p-value. Data are presented as mean ± SEM.

To validate the bioinformatic RNA-Seq results, we used RT-qPCR to analyze the mRNA levels of the four opioid receptor genes and the *Cnr1* gene in L3/L4 DRG samples from male mice 28 days after SNI surgery. As shown in Fig. 3a, nerve injury significantly decreased the expression of *Oprm1*, *Oprd1*, *Oprk1*, *Oprl1*, and *Cnr1* transcripts, validating the RNA-Seq results.

### REST is present at the *Oprm1* and *Cnr1* promoter regions in the injured DRG of wild-type mice

We used publicly available bioinformatic data from the geneXplain platform (https://genexplain.com/genexplain-platform/) to identify potential REST binding sites on the promoter regions of *Oprm1* and *Cnr1*. To determine whether nerve injury affects REST binding at these promoters, we performed chromatin immunoprecipitation (ChIP)-qPCR analysis on L3/L4 DRG samples from male mice 28 days after SNI (Fig. 3b). SNI significantly increased the presence of REST on both the *Oprm1* and *Cnr1* promoters in the mouse DRG. The ChIP data corroborated our RT-qPCR results, suggesting that REST in the DRG may play a role in repressing *Oprm1* and *Cnr1* transcript levels in wild-type mice following nerve injury.

### DRG neuron-specific *Rest* cKO attenuates pain hypersensitivity induced by nerve injury in mice

**Fig. 4:**
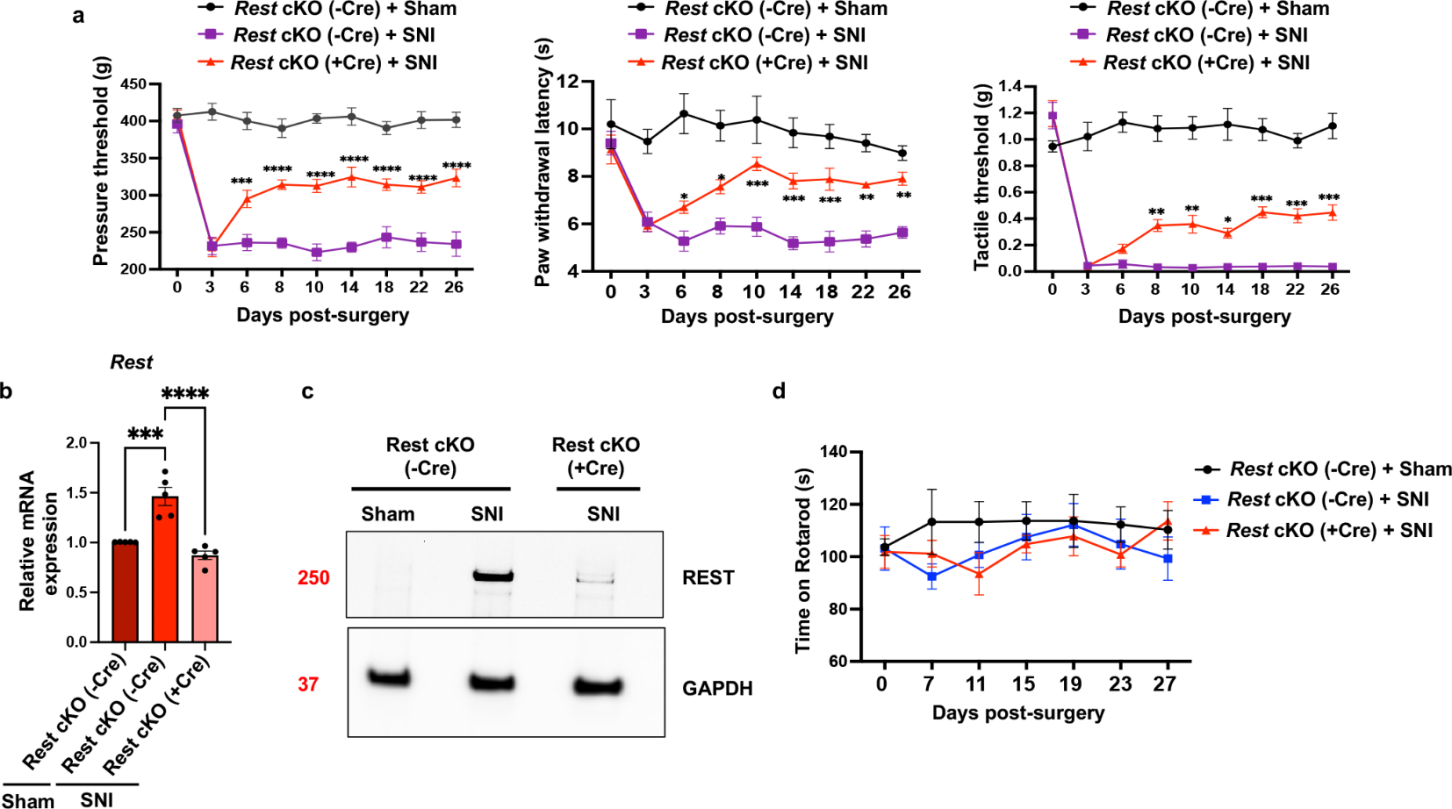
(**a**) DRG neuron-specific *Rest* cKO attenuates nerve injury-induced pain hypersensitivity in mice. Nerve injury-induced pain hypersensitivity was assessed in *Rest* cKO mice as described in Fig. 1. The mouse groups were: *Rest* cKO (-Cre) + sham, *Rest* cKO (-Cre) + SNI, and *Rest* cKO (+ Cre) + SNI. *N* = 6 per group. A two-way ANOVA followed by Tukey’s multiple comparison test was used. P-values are shown compared with *Rest* cKO (-Cre) + SNI group. Data are presented as mean ± SEM. (**b**–**d**) SNI results in decreased mRNA and protein levels of the *Rest* gene in the injured DRG of *Rest* cKO mice compared with their wild type littermates, but it does not cause significant changes in the rotarod assays. (**b**) mRNA samples from the L3/L4 DRG tissues of mice, as characterized in Fig. 4a, were analyzed. The mouse groups were: *Rest* cKO (-Cre) + sham, *Rest* cKO (-Cre) + SNI, and *Rest* cKO (+ Cre) + SNI. RT-qPCR analysis was used to measure the levels of *Rest* transcripts. *Gapdh* was used as an internal control. A two-way ANOVA followed by Tukey’s multiple comparison test was used. Data are presented as mean ± SEM. *N* = 5 mice per group. P-values are shown. (**c**) Western blotting assays from the L3/L4 DRG tissues to detect REST protein. GAPDH was used as an internal control. Approximate molecular weights in kilodalton are also shown. (**d**) Rotarod assays of *Rest* cKO mice indicated no significant difference between sham and SNI treatment.

Next, we used full-length *Rest* cKO mice^22^ to determine whether the increased expression of *Rest* in DRG neurons plays a causal role in the development of chronic neuropathic pain. We performed SNI or sham surgery on male *Rest* cKO (+ Cre) and their control littermates, Rest cKO (-Cre). We measured pressure, heat, and tactile withdrawal thresholds in the ipsilateral hindpaws over 4 weeks, as we did in the wild-type mice. As shown in Fig. 4a, SNI significantly reduced the pressure, thermal, and tactile withdrawal thresholds in the ipsilateral hindpaw of *Rest* cKO (-Cre) mice compared with control *Rest* cKO (-Cre) + sham mice. The Rest cKO (+ Cre) + sham mice exhibited behavior similar to that of the Rest cKO (-Cre) + sham mice, and the data for Rest cKO (+ Cre) + sham were omitted for simplicity. We then compared SNI-induced behaviors between *Rest* cKO (-Cre) and *Rest* cKO (+ Cre) mice. The data indicated that the withdrawal thresholds of the ipsilateral hindpaw did not differ significantly between these two groups during the first 3 days after SNI (the acute phase of pain). However, SNI-induced hypersensitivities were markedly attenuated in *Rest* cKO (+ Cre) mice from 6 to 26 days after SNI (the transition to the chronic phase of pain). These results were consistent with those observed using a different strain of *Rest* cKO (+ Cre) mice^24^.

To ascertain the status of REST transcript and protein expression levels in these mice, we performed RT-qPCR using L3/L4 DRG tissues collected 28 days after either SNI or sham surgery. As shown in Fig. 4b, SNI increased *Rest* transcript levels in *Rest* cKO (-Cre) mice compared to sham surgery, similar to what we observed in wild-type mice (Fig. 1). Furthermore, *Rest* transcript levels in the DRG were significantly lower in *Rest* cKO (+ Cre) + SNI mice compared to *Rest* cKO (-Cre) + SNI mice. However, we did not observe a complete elimination of *Rest* transcripts in the DRG of *Rest* cKO (+ Cre) + SNI mice (see below). We also performed a Western blotting assay with the corresponding tissues to evaluate REST protein levels (Fig. 4c; raw data for the Western blotting assays is shown in Supplementary Fig. 2). SNI caused an increase in REST protein levels in *Rest* cKO (-Cre) mice compared with sham surgery, corroborating our current findings in wild-type mice (Fig. 1c) and previous studies^24^. SNI produced significantly lower levels of REST in *Rest* cKO (+ Cre) mice compared to *Rest* cKO (-Cre) mice. However, there were still low levels of residual REST protein present in *Rest* cKO (+ Cre) mice (see Discussion). To further assess the level of *Rest* transcripts remaining in *Rest* cKO (+ Cre) mice, we reanalyzed the RT-qPCR data, including the *Rest* cKO (+ Cre) + sham group (Supplementary Fig. 3). The data indicated that the residual *Rest* transcript levels in *Rest* cKO (+ Cre) + sham mice were approximately 50% of those in *Rest* cKO (-Cre) + sham mice. The level of residual REST expression observed in our experiments is consistent with previous observations reported using a different *Rest* cKO mouse line^25,24^; see Discussion).

We then performed rotarod tests to determine whether SNI induced impairment of motor functions. As shown in Fig. 4d, there were no significant differences in rotarod performance among *Rest* cKO (-Cre) + sham, *Rest* cKO (-Cre) + SNI, or *Rest* cKO (+ Cre) + SNI mice. This suggests that SNI did not impair motor functions.

### **DRG neuron-specific *Rest*cKO rescues the SNI-induced reduction in *Oprd1 *and *Cnr1 *expression in the DRG of mice.**

**Fig. 5:**
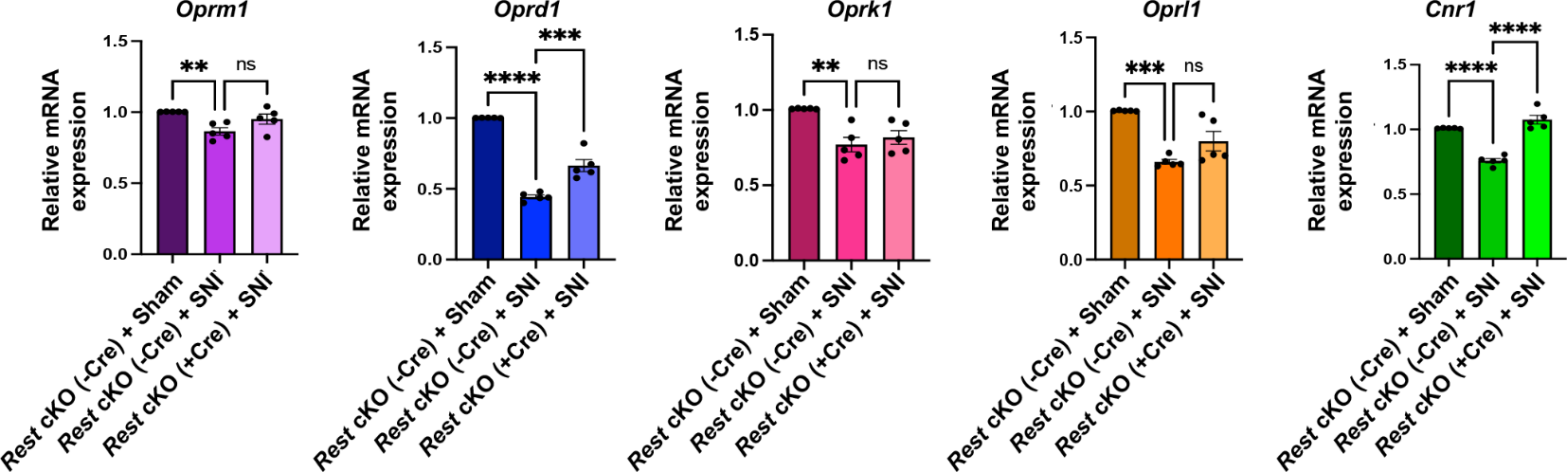
DRG neuron-specific *Rest* cKO rescues the SNI-induced reduction in the expression of *Oprd1* and *Cnr1* in the DRG of mice. mRNA samples from the L3/L4 DRG tissues of mice, characterized in Fig. 4, were used for RT-qPCR analysis of *Oprm1*, *Oprd1*, *Oprk1*, *Oprl1*, and *Cnr1* transcripts. *Gapdh* was used as an internal control. Data from *Rest* cKO (-Cre) + sham, *Rest* cKO (-Cre) + SNI, and *Rest* cKO (+ Cre) + SNI are shown. A two-way ANOVA followed by Tukey’s multiple comparison test was used. Data are presented as mean ± SEM. P-values are shown. *N* = 5 mice per group.

We then investigated the role of REST in DRG neurons in the SNI-induced downregulation of opioid and cannabinoid receptor genes in *Rest* cKO (+ Cre) mice. We performed RT-qPCR using L3/L4 DRG tissues from *Rest* cKO (-Cre) + sham, *Rest* cKO (-Cre) + SNI, and *Rest* cKO (+ Cre) + SNI mice. As shown in Fig. 5, SNI in male *Rest* cKO (-Cre) controls decreased the expression of *Oprm1*, *Oprd1*, *Oprk1*, *Oprl1*, and *Cnr1* compared to sham surgery, recapitulating the findings observed in wild-type mice (Fig. 3). Remarkably, the diminished expression levels of *Oprd1* and *Cnr1* in control *Rest* cKO (-Cre) + SNI mice were significantly rescued in *Rest* cKO (+ Cre) + SNI mice, suggesting that REST regulates these genes. However, the expression levels of *Oprm1*, *Oprk1*, and *Oprl1* in the DRG did not differ significantly between *Rest* cKO (-Cre) + SNI and *Rest* cKO (+ Cre) + SNI mice. These results suggest that nerve injury silences *Oprd1* and *Cnr1* genes via REST in primary sensory neurons of mice.

## Discussion

Our findings indicate that nerve injury suppresses the transcription of *Oprd1* and *Cnr1* through REST in primary sensory neurons. *Cnr1* expression levels in DRG neurons are important for regulating pain hypersensitivity; *Cnr1* cKO in DRG neurons causes pain hypersensitivity in mice^30^. Similarly, we observed that nerve injury diminishes *Cnr1* expression in the DRG via G9a^19^, a cofactor of REST. We found that *Rest* cKO similarly attenuated thermal, pressure, and touch hypersensitivities in the DRG of nerve-injured mice. This decrease in *Cnr1* expression also corresponded to the presence of REST protein on the *Cnr1* gene chromatin in the injured DRG. These results suggest that the REST-G9a complex is involved in this process.

*Oprd1* cKO in DRG neurons increases pain hypersensitivity caused by nerve injury or tissue inflammation in mice, indicating that DOR expression levels in DRG neurons normally help restrain pain hypersensitivity^31,17^. Since there is no apparent RE1-binding site at the *Oprd1* promoter, REST likely regulates DOR expression in DRG neurons indirectly—potentially by altering the expression of a transcription factor that binds to the *Oprd1* promoter or via one or more microRNAs. Another possibility is that REST does bind to the *Oprd1* promoter, but current methodologies may not be sufficient to detect such binding. A genome-wide ChIP-sequencing approach could help address this issue.

We are not aware of any DRG cKO studies involving *Oprk1* and *Oprl1*. However, constitutive KO of *Oprk1* in mice has been shown to enhance sensitivity to chemically induced acute visceral pain^32^. In contrast, *Oprl1* KO mice do not exhibit differences in nociceptive thresholds compared to wild-type animals^33,34^. These studies suggest that nerve injury-induced upregulation of REST likely promotes pain hypersensitivity primarily through the downregulation of *Oprd1* and *Cnr1* in DRG neurons.

Our *Rest* cKO (+ Cre) mice exhibited about 50% residual REST levels compared with *Rest* cKO (-Cre) mice. As described in the Results section, similar levels of residual REST expression were also observed in previous studies using a different *Rest* cKO mouse line^25,24^. One possible reason for these results is that Advillin-Cre–induced target gene knockout does not occur in 100% of DRG neurons^35^. The remaining REST levels suggest that we can only detect low-affinity REST targets in our system (e.g., *Oprd1* and *Cnr1*). In contrast, high-affinity targets may be repressed by the residual REST and thus remain undetectable compared to the control. This might explain the lack of upregulation of the *Oprm1* gene in *Rest* cKO (+ Cre) + SNI mice compared with *Rest* cKO (-Cre) + SNI mice (Fig. 5), despite REST being present on the *Oprm1* promoter elements (Fig. 3).

Our studies also indicated that *Rest* transcript levels did not consistently correspond to REST protein levels (Fig. 4, b versus c). A similar discrepancy in REST expression levels was observed previously in a different *Rest* cKO mouse line^24^. This suggests that REST may be regulated not only at the transcriptional level but also at the protein degradation level, as we and others have proposed in neural tissues^36,37^.

Because this study only involved nerve injury-induced chronic pain in male mice, we could not compare our results with those from female mice. However, it is important to note that studies have indicated sexual dimorphism in the nerve injury-induced expression of the pain-related gene^38,39^. Additionally, sex hormones are known to affect *Oprm1* gene chromatin in female mice^40^. This suggests that morphine-induced analgesia might be more effective in the DRG of female mice compared to male mice. Interestingly, one study found that women used lower dosages of opioids than men after surgery^41^. However, several confounding factors could account for this observation. Reports of sex differences in opioid analgesia have been controversial^42,43^. For example, studies on µ-opioids indicated that there was no significant association between sex and analgesia. However, results differed when examining patient-controlled morphine analgesia studies^44^. In addition, as previously reported, the temporal and spatial expression of the *Oprm1* gene in primary afferent neurons following spinal nerve injury in a rat experimental model was found to be dynamic and time-dependent^45^.

Since *Rest* cKO (+ Cre) in DRG neurons attenuates SNI-induced pain hypersensitivity and the reduction in the expression of *Oprd1* and *Cnr1*, it is conceivable that inhibiting REST activity could reduce chronic neuropathic pain and enhance opioid/cannabinoid analgesic effects by increasing the expression of *Oprd1* and *Cnr1* genes in DRG neurons. Therefore, REST might be a valuable target for treating neuropathic pain.

## Methods

### Mice

All mouse experiments were approved by the Institutional Animal Care and Use Committee (IACUC) of The University of Texas MD Anderson Cancer Center (MDACC). All methods were performed in accordance with the relevant guidelines and regulations of MDACC. We used C57BL/6 wild-type male mice obtained from the Research Animal Support Facility at The University of Texas MD Anderson Cancer Center.

Wild-type C57BL/6 mice were purchased from in-house MDAnderson facility (Experimental Radiation Oncology) and then bred in our mouse colony. *Rest* conditional full-length KO (obtained from Dr. Gail Mandel^22^), and characterized by measuring transcript and protein levels of the *Rest* gene in littermates (-Cre) and cKO (+ Cre) mice. Deletion of *Rest* in mouse DRG neurons was generated by crossing female *Rest*-loxP^+/+^ mice and male Advillin-Cre mice (purchased form The Jackson Laboratory, Bar Harbor, ME, #032536). Advillin-Cre^+/−^ : *Rest*-loxP^+/−^ mice obtained from the first generation were crossed with female *Rest*-loxP^+/+^ mice to get Advillin-Cre^+/−^ : *Rest*-loxP^+/+^ mice (*Rest*-cKO mice). Littermates without Cre expression (Advillin-Cre^−/−^ : *Rest*-loxP^+/+^) were used as controls. Mice were earmarked at the time of weaning (3 weeks after birth), and tail biopsies were used for polymerase chain reaction genotyping.

The primers used for genotyping Advillin-Cre^+/−^ were: Avil/003: CCCTGTTCACTGTGAGTAGG; Avil/002: AGTATCTGGTAGGTGCTTCCAG; Cre/01:GCGATCCCTGAACATGTCCATC. The wild-type allele produces a 500-bp product, and the mutant allele produces a 180-bp product. For genotyping *Rest-GTi/ GTi*, the primers used were: GTA5: TGGATGTTGAGGTCCGTTGTG, GTB5: GCTACGGATCCCTTCTTCCC, and GTB1: AACGGCCCCCGACGTCCCTGG to produce a wild-type product of 480 bp and a mutant product of 600 bp. In addition, Western blotting assays indicated substantial loss of REST protein in the DRG of male *Rest* cKO mice compared to those of their littermates.

Male mice (*N* = 6–8 per group; 8–10 weeks of age) were used for the final experiments. To improve the breeding performance of *Rest* cKO mice, DietGel Prenatal supplement (ClearH_2_O, Inc., Westbrook, ME, #69-503-02) was given as a feed additive to the breeding pairs (10 g per mouse twice weekly).

### Spared nerve injury

SNI surgery was performed as we and others have described previously^46,7^. Mice were anesthetized with 2–3% isoflurane, and the sciatic nerve and its 3 terminal branches (the sural, common peroneal, and tibial nerves) of the left leg were exposed under a surgical microscope. The tibial and common peroneal nerves were tightly ligated with a 6 − 0 silk suture and sectioned distal to the ligation sites, leaving the sural nerve intact. For postsurgical therapy, mice received a single dose of the local anesthetic Bupivacaine (0.5% Bupivacaine, 50 µl) at the surgical site. No additional analgesics or antibiotics were administered to the mice. To aid in recovery, mice were given supplemental warmth by using heating pads. Following the surgery, the animals were closely monitored by for 2–3 h to ensure a full recovery. Mice were then monitored every day for 3 days post-surgery.

### Nociceptive behavior test

We used hindpaw withdrawal thresholds in response to a noxious pressure stimulus (mechanical hyperalgesia), a noxious radiant heat stimulus (thermal hyperalgesia), and von Frey filaments (tactile allodynia) to assess pain, as we have published previously^17,24^. To familiarize the mice with the testing conditions and environments, they were kept on the wired metal grid and thermal testing apparatus for 30 min each day for 3 days before testing began.

A Rodent Pincher analgesia meter (IITC Life Science) was used to measure the pressure thresholds in the mice. The hindpaw was put between the 2 arms of the pincher, and steady pressure was applied at increasing intensity until the mouse showed a pain response, as indicated by paw withdrawal and/or vocalization. The force in “*g*” that elicited the pain response was recorded. Each trial was repeated 3 times with an interval of 2 min in between, and the mean value was recorded.

For thermal sensitivity, the plantar test (Hargreaves method) with heated glass (IITC Life Science, Woodland Hills, CA) was used. The glass surface was maintained at 30 °C, and the active intensity of the radiant lamp was maintained at 30%. Mice were acclimatized for 30 min in the observation chamber before testing. The planar surface was heated by a mobile radiant heat source, and the time of paw withdrawal was recorded by an automatic time recorder associated with the apparatus. Each trial was repeated 3 times, and the mean time to withdrawal was calculated.

For the tactile sensitivity testing, the mice were habituated on the wired metal grid for 30 min immediately before the test. The von Frey filament (Stoelting, Wood Dale, IL) was applied to the planar surface of the hindpaw for 3–5 s. Brisk withdrawal or paw licking was considered a positive response. The filament force value was increased until a positive response was detected and then decreased to the next lower value. The 50% likelihood of withdrawal was calculated using the up-down von Frey method^47^.

### Rotarod test

The rotarod apparatus (Panlab Harvard Apparatus, Cornella, Spain) was set up in acceleration mode with a range of 4 to 40 rpm such that the maximum speed was reached in 300 s. The mice were trained 3 times a day for 5 min each for 3 days to learn the task. On test day, the mice were gently placed on the rotarod, and the rotarod was started at the initial speed of 4 rpm. The speed was increased until the mice fell from the moving rotarod, at which point the time was recorded. The trial was repeated 3 times for each mouse, and the mean time was recorded.

### Tissue collection

After the nociceptive and rotarod testing, the mice were deeply anesthetized using 3–4% isoflurane and humanely killed. The L3 and L4 DRG tissues were immediately collected after euthanasia by using the hydraulic extrusion method described earlier^48^.

### RNA-Sequencing

Total RNAs were extracted from the L3 and L4 DRG tissues using TRIzol (Thermo Fisher Scientific, #15596026). The RNA concentration was quantified using a NanoDrop 1000 (Thermo Fisher Scientific). Good quality of RNA was confirmed using 260 nm/280nm absorbance of 2 using a Nanodrop spectrophotometer and was then sent to the Advanced Technology Genomics Core at MD Anderson for RNA-sequencing. We trimmed the RNA sequencing data for low-quality reads using trim_galore, then mapped using STAR onto the mouse genome build mm10. Gene expression was determined using feature Counts^49^. Differentially expressed genes were determined after normalization with Remove Unwanted Variation^50^ using the EdgeR R package. Enriched pathways were inferred using Gene Set Enrichment Analysis (GSEA) (Subramanian et al., 2005) against pathway compendia REACTOME and Gene Ontology Biological Processes as compiled by the Molecular Signatures Database (MSigDB)^51^. Pathways were considered significant at a false discovery rate < 0.25 per the GSEA developer’s best practices. RNA-Seq data can be accessed from the GEO website submission can be accessed through the GEO data repository (https://www.ncbi.nlm.nih.gov/geo/) using accession # GSE263828 (Reviewer token: gnirugioznklpgp).

### Quantitative RT-PCR

One microgram of RNA was first treated with RNAase-free DNase and then used for reverse transcription with the SuperScript IV VILO Master Mix reverse transcription kit (11766050; Thermo Fisher Scientific). Two microliters of complementary DNA was diluted 5 times and added to a 20-µL reaction volume with SYBR Green real-time PCR mixture (Thermo Fisher Scientific, #A25778). Real-time PCR was performed using the QuantStudio 6 Flex real-time PCR system (Applied Biosystems, Waltham, MA). The thermal cycling conditions were as follows per the manufacturer’s instructions: 1 cycle at 50 °C for 2 min, 1 cycle at 95 °C for 2 min, and 40 cycles at 95 °C for 15 s and at 60 °C for 45 s. The primers used are shown in Table 1.

**Table 1:** List of primers used in qRT-PCR assays.

*Gapdh* Forward	TTCACCACCATGGAGAAGGC	^52^
*Gapdh* Reverse	GGCATGGACTGTGGTCATGA	
*Rest* Forward	CAAGTGCAACTACTTCTCAGACAGA	^22^
*Rest* Reverse	AAGGACAAAGTTCACATTTATACGG	
*Oprm1* Forward	GATCCTCTCTTCTGCCATTGGTC	OriGene Technologies, Inc, MP210417
*Oprm1* Reverse	TGAGCAGGTTCTCCCAGTACCA	
*Oprd1* Forward	CCATCATGGTCATGGCAGTGAC	OriGene Technologies, Inc, MP210415
*Oprd1* Reverse	CGAAGGCAAAGAGGAACACGCA	
*Oprk1* Forward	ATCACCGCTGTCTACTCTGTGG	OriGene Technologies, Inc, MP210416
*Oprk1* Reverse	GTGGTAGTAACCAAAGCATCTGC	
*Oprl1* Forward	GCTCAGCACAAGTGGAGGATGA	OriGene Technologies, Inc, MP210419
*Oprl1* Reverse	GGCTGTAGCAGACAGAGATGATC	
*Cnr1* Forward	ATCGGAGTCACCAGTGTGCTGT	^53^
*Cnr1* Reverse	CCTTGCCATCTTCTGAGGTGTG	

### Western blotting

Protein lysate was made from L3/L4 DRGs using RIPA buffer (89900, Thermo Scientific) added with protease and phosphatase inhibitor (78440, Thermo Scientific). Protein concentration was determined by BCA kit (23228, Thermo Scientific). 20 ugm of protein was loaded and separated on NuPAGE 4 to 12%, Bis-Tris gel (NP0335BOX, Thermo Scientific) under reducing condition in MES SDS running buffer (NP002, Thermo Scientific) as per manufacturer recommendation. Running time was 45 min at 200 V. Transfer was done on nitrocellulose membrane (1620112, Bio Rad) for 90 min at 120 V. The membrane was blocked by 5% skim milk solution prepared by 0.05% Tween 20 in Tris Buffer Saline (TBST) for 1 h, The primary antibodies were probed for overnight at 4 °C. Membrane washed with TBST for 3 times each and incubated with IRDye 800CW (926-32213, 1:15000, Licor ) and IRDye 680RD (926-68072, 1:15000,Licor) for 1 h. After 3 washes with TBST, membrane was observed using ChemiDOC MP imaging system (Biorad). Rabbit Anti-REST (#07-579, 1:250, Millipore) and mouse Anti GAPDH (MAB374, 1:300, Millipore) control antibodies were used to probe the membrane.

### ChIP assays

Match tool in the geneXplain platform (https://genexplain.com/genexplain-platform/), which uses the TRANSFAC 2.0 database (version 2023.2), was used to predict potential REST binding sites in the genome based on the positional weight matrices of binding sites in the database. The promoters of mouse *Oprm1* and *Cnr1* genes were screened from − 1000 bp to + 1000 bp relative to the transcriptional start site. Next, ChIP-qPCR primers were designed using the NCBI Primer-BLAST tool (https://www.ncbi.nlm.nih.gov/tools/primer-blast/).

L3/L4 DRG tissues from 5 animals (10 DRGs) per group were collected. qChIP was performed using Covaris truChIP (#520237) and Cell Signaling SimpleChIP (#56383) kits as described by the manufacturer with some modifications. Briefly, DRGs from both sham and SNI groups (*n* = 10 from 5 mice, L3 and L4) were crosslinked by 1X fixing buffer A (Covaris truChIP #520237) and paraformaldehyde for 10 min. The nuclear fraction was isolated and subjected to sonication (Diagenode Bioruptor PICO B01080010) to break the DNA into 200–700 bp fragments (4 cycles 30/30 on/off time, easy mode; Supplementary Fig. 4). After fragmentation, the chromatin was incubated with either 15 µg REST (Millipore, #07-579 lot#3455213) or 1 µg IgG (Sigma, #27290) antibodies overnight at 4 °C with rotation. The eluted protein-DNA mixture was de-crosslinked using 5 M NaCl and Proteinase K overnight at 65 °C on a Thermomixer. Finally, the DNA was purified using a Qiagen PCR purification kit (#28104) according to the manufacturer’s protocol. The DNA was then subjected to qRT-PCR assay with the primers listed in Table 2.

**Table 2:** List of primers used in ChIP assays.

	5’-Sequence-3’
Chip-m*Oprm1*-F	CCGCAGCAAGCATTCAGAAC
Chip-m*Oprm1*-R	TGCCATCAACGTGGGACAAG
Chip-m*Cnr1*-F	GGTCGTTGGTGGCAAAGAG
Chip-m*Cnr1*-R	GCTCCAAACTTTGCTGCG

Student *t* test was used to compare the groups. Data were expressed as mean ± SEM.

## Supplementary Information

Supplementary Information.

## Data Availability

The datasets generated and/or analyzed during the current study are available in the GEO repository (https://www.ncbi.nlm.nih.gov/geo/) using accession # GSE263828 (Reviewer token: gnirugioznklpgp).
